# Head and Neck Cancer: A Potential Risk Factor for Parkinson’s Disease?

**DOI:** 10.3390/cancers16132486

**Published:** 2024-07-08

**Authors:** Il Hwan Lee, Dong-Kyu Kim

**Affiliations:** 1 Department of Otorhinolaryngology-Head and Neck Surgery, Chuncheon Sacred Heart Hospital, Hallym University College of Medicine, Chuncheon 24252, Republic of Korea; 2 Institute of New Frontier Research, Division of Big Data and Artificial Intelligence, Chuncheon Sacred Heart Hospital, Hallym University College of Medicine, Chuncheon 24252, Republic of Korea

**Keywords:** cancer, head, neck, Parkinson’s disease, cohort study

## Abstract

**Simple Summary:**

This study revealed an increased incidence of Parkinson’s disease (PD) events in individuals diagnosed with head and neck cancer (HNC). The statistically significant rise in PD risk became evident after seven years post HNC diagnosis, with the elevated risk persisting after that. Particularly noteworthy was the heightened association between PD events and middle-aged patients with HNC as well as cases where the malignancies were localized to the oral cavity. As a result, we emphasize the importance of implementing proactive strategies for early detection and management in patients with HNC to address this elevated risk effectively.

**Abstract:**

Head and neck cancers (HNC) are frequently associated with neurodegeneration. However, the association between HNC and Parkinson’s disease (PD) remains unclear. This study aimed to clarify the relationship between HNC and subsequent PD. This retrospective study used data from a nationally representative cohort. Patients with HNC were identified based on the presence of corresponding diagnostic codes. Participants without cancer were selected using 4:1 propensity score matching based on sociodemographic factors and year of enrollment; 2296 individuals without HNC and 574 individuals with HNC were included in the study. Hazard ratios (HR) for the incidence of PD in patients with HNC were calculated using 95% confidence intervals (CI). The incidence of PD was 4.17 and 2.18 per 1000 person-years in the HNC and control groups, respectively (adjusted HR = 1.89, 95% CI = 1.08–3.33). The HNC group also showed an increased risk of subsequent PD development. The risk of PD was higher in middle-aged (55–69 years) patients with HNC and oral cavity cancer. Our findings suggest that middle-aged patients with HNC have an increased incidence of PD, specifically those with oral cavity cancer. Therefore, our findings provide new insights into the development of PD in patients with HNC.

## 1. Introduction

Head and neck cancer (HNC) refers to a group of biologically similar cancers that originate in the tissues and organs of the head and neck region. It can develop in various regions, including the oral cavity, pharynx, larynx, paranasal sinuses, and neck [1]. In South Korea, the incidence rate of HNC is reported to be 5–6 per 100,000 person-years, which is lower than that of other cancers. However, the mortality rate of HNC remains notable, at 1.5 per 100,000 person-years [2]. Recent advancements in cancer diagnostic technologies and essential therapeutic interventions such as surgical resection, radiation therapy, and chemotherapy have significantly extended the survival of patients with HNC. Despite these improvements, increased survival rates have led to an increased incidence of late-onset complications. Among these, long-term adverse effects including neurodegenerative diseases are a growing concern. This condition may occur as a result of the malignancy itself or its treatment and often appears months or years after treatment. Neurodegenerative sequelae associated with HNC can severely compromise the quality of life of patients and their families and impose a substantial burden on the healthcare system. Therefore, understanding and addressing the long-term neurodegenerative effects of HNC and its treatments are crucial for improving patient outcomes and mitigating healthcare challenges.

Parkinson’s disease (PD) is one of the most common neurodegenerative movement disorders caused by the premature death of dopaminergic neurons in the substantia nigra of the midbrain [3]. This disease leads to symptoms such as tremors, rigidity, bradykinesia, and postural instability. Although its pathological characteristics are well defined, the underlying causes and mechanisms of neuronal death remain unclear. Recent studies have reported on the association between cancer and neurodegenerative diseases, with conflicting views on the relationship between various cancers and PD [4,5,6]. Some studies have shown that PD occurs less frequently in patients with smoking-related cancers than in controls, indicating an inverse association [7]. In contrast, other studies reported a higher incidence of PD in patients with melanoma than in the general population [8]. Although previous studies have explored the association between various malignancies and PD, no studies have investigated the relationship between HNC and PD.

This study aimed to elucidate the association between HNC and PD. We investigated the potential risk of PD in patients diagnosed with HNC using National Sample Cohort data from the Korean National Health Insurance Service. The comprehensive nature of this dataset, which included a wide spectrum of diseases across the nationwide population, allowed us to explore the potential links between these two conditions. Furthermore, we rigorously controlled for potential confounding variables including clinical status and demographic factors to enhance the validity of our results.

## 2. Materials and Methods

### 2.1. Study Design

**Figure 1:**
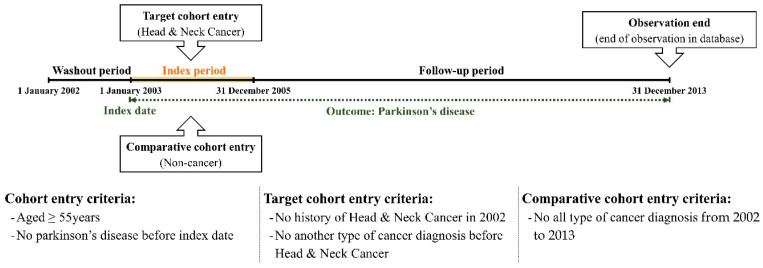
Description of the study design.

**Figure 2:**
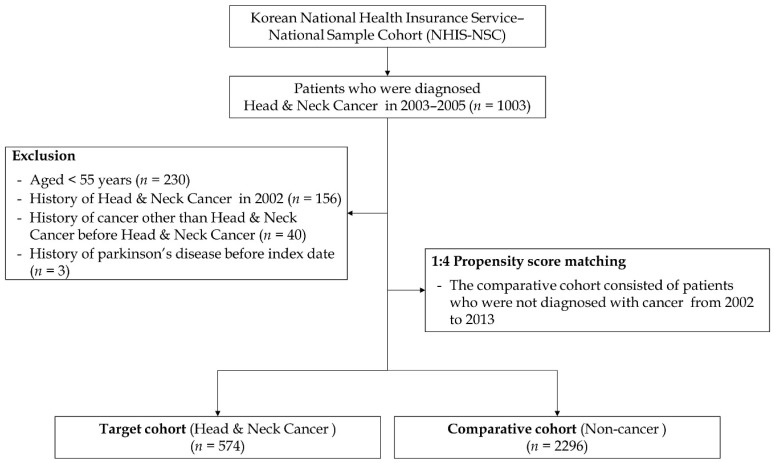
Flow chart of study enrollment.

We excluded the first year (January to December 2002) from the cohort dataset as a washout period to eliminate the risk of PD prior to the diagnosis of HNC. Subsequently, we enrolled patients diagnosed with HNC during the index period (2003–2005). The cancer group was defined by the presence of diagnostic codes for HNC (C00–C14 and C30–C32). We included individuals who had these diagnostic codes more than twice within the index period or who had inpatient hospitalization with these diagnostic codes. To enhance the accuracy of the outcome, we excluded patients under 55 years of age, those who died during the index period, or those diagnosed with dementia before the cancer diagnosis. We then selected control participants as a comparison group using a propensity score-matching methodology, selecting four participants without cancer for each patient with cancer. During propensity score matching, we matched participants based on all independent variables and year of enrollment (cancer diagnosis). In this study, we controlled for several independent variables in the target and comparative cohorts to refine the outcomes. Therefore, we selected multiple independent variables including age, sex, residence, household income, and comorbidities. Comorbidities were adjusted using the Charlson comorbidity index (CCI), a weighted index used to classify patient comorbidities. The primary endpoint of this study was defined as the occurrence of PD (G20) events until the final date of the cohort dataset. If the patients did not experience these events by the end of the follow-up period, they were censored at that time point. The study design and participant enrollment are illustrated in Figure 1 and Figure 2. 

### 2.2. Study Population

Our nationwide representative cohort dataset, spanning from 2002 to 2013, encompasses a comprehensive collection of healthcare data for 1,025,340 adults sourced from the South Korean healthcare claims database. This dataset is meticulously structured to include a wide array of healthcare information, capturing both inpatient and outpatient visits, various medical procedures, and prescription records. The diagnostic codes employed in this dataset adhere to the International Classification of Diseases, 10th Revision, Clinical Modification (ICD-10-CM) standards, ensuring consistency and accuracy in disease classification. Each individual within this dataset is assigned a unique identification number at birth. This unique identifier plays a crucial role in maintaining the integrity of the dataset by preventing the omission or duplication of healthcare claims data. As a result, our dataset provides an accurate and reliable reflection of the entire South Korean population over the study period, significantly minimizing the potential for selection bias. The dataset’s robustness is further highlighted by its ability to track healthcare utilization and outcomes comprehensively, allowing for detailed analyses of health trends and patterns within the population. It offers valuable insights into the prevalence of various diseases, the effectiveness of treatments, and the healthcare behaviors of individuals. By encompassing a diverse and representative sample, this dataset supports a wide range of epidemiological studies and health policy assessments, thereby contributing to the improvement of public health strategies and interventions in South Korea. Moreover, the longitudinal nature of the dataset enables researchers to conduct long-term studies, examining changes in health status and healthcare utilization over time. This is particularly beneficial for identifying trends and making informed predictions about future healthcare needs. The high level of detail and extensive coverage of the dataset make it an invaluable resource for healthcare researchers, policymakers, and practitioners aiming to enhance the quality and efficiency of healthcare delivery in South Korea. In summary, our nationwide representative cohort dataset from 2002 to 2013 stands as a highly reliable and comprehensive source of healthcare information. This cohort study utilized a nationwide population-based dataset derived from the national health claims data collected by the Korean National Health Insurance Service. The dataset was obtained following a de-identification process. The study was approved by the Institutional Review Board (IRB) of Hallym Medical University, Chuncheon Sacred Hospital (IRB No. 2021-08-006), which waived the requirement for written informed consent due to the de-identified nature of the data. Owing to government policies, the original datasets are not publicly accessible.

### 2.3. Statistical Analysis

The incidence rate was determined to assess the frequency of disease or other incidents over a given period reported per 1000 person-years. Person-years were calculated for the following three scenarios: (1) for cases of death, the period from the initial cancer diagnosis to the date of death; (2) for specific events, the duration from the initial cancer diagnosis to the date of the first occurrence of these events; and (3) if no events occurred, the period from the initial cancer diagnosis to the end of the study. To evaluate whether patients with cancer have an increased risk of developing specific diseases, we employed Cox proportional hazards regression analyses to compute the hazard ratio (HR) and 95% confidence interval (CI) after adjusting for other independent variables. All statistical analyses were performed using R software (version 3.5.0; R Foundation for Statistical Computing, Vienna, Austria). Statistical significance was set at *p* < 0.05. 

## 3. Results

### 3.1. General Characteristics of the Cohort

**Figure 3:**
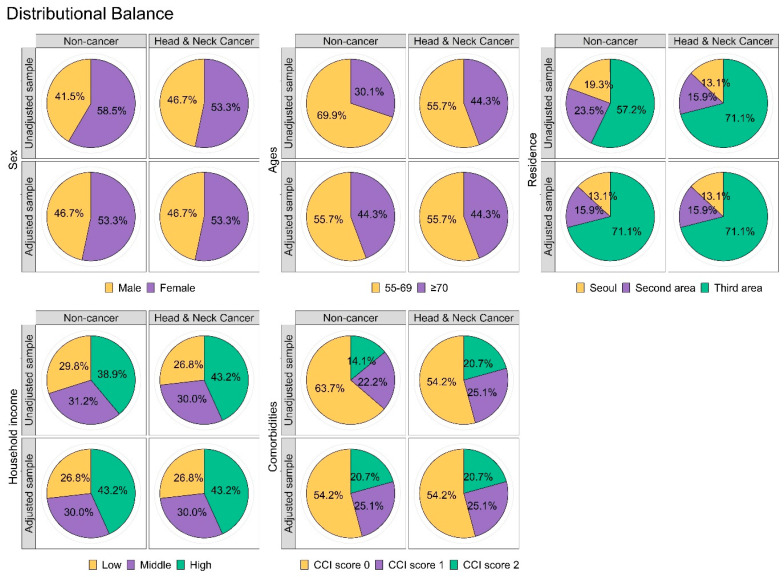
Confirmation of propensity scoring matching using the balance plot for all independent variables.

The HNC and non-cancer groups were similarly distributed with respect to all covariates used for sample matching. In addition, we found no significant differences in any of the independent variables between the two cohorts (Table 1). We also performed a balance plot test to confirm appropriate matching (Figure 3).

### 3.2. Incidence Rate of PD

To investigate the incidence rate of PD, we conducted an analysis that included 19,301.1 person-years in the non-cancer group and 4077.7 person-years in the HNC group. Our findings revealed a notable difference in the incidence rates between the two groups. Specifically, the incidence of PD in the HNC group was 4.17 per 1000 person-years. In contrast, the non-cancer group exhibited a lower incidence rate of 2.18 per 1000 person-years. These results, as detailed in Table 2, suggest a higher incidence of PD among individuals with a history of HNC compared to those without cancer. This significant disparity underscores the importance of further research to explore the potential links and underlying mechanisms between HNC and the increased risk of developing PD. By analyzing these incidence rates, we can better understand the health outcomes and risks associated with HNC, guiding future studies and potential interventions.

### 3.3. Risk Rate of PD

We conducted an analysis of the HR for PD events using both univariate and multivariate Cox regression models over a 10-year follow-up period, as presented in Table 2. This analysis allowed us to assess the impact of HNC on the onset of PD while controlling for various covariates. After adjusting for all covariates, our results demonstrated a significant association between HNC and the onset of PD events, with an adjusted HR of 1.89 (95% CI = 1.08–3.33). This indicates that individuals with HNC have an 89% higher risk of developing PD compared to those without HNC, after accounting for other influencing factors. Moreover, our evaluation of the risk of developing PD throughout the follow-up period revealed critical insights. Notably, the risk was found to be particularly high in the seventh year following the diagnosis of HNC (as shown in Table 3). Although the risk diminished after the seventh year, a significant risk ratio for developing PD persisted throughout the entire follow-up period. This prolonged elevated risk underscores the need for ongoing monitoring and preventive strategies for PD in patients with a history of HNC. The findings of this analysis highlight the importance of long-term follow-up and comprehensive management strategies for patients diagnosed with HNC. The increased risk of PD observed in these patients suggests potential underlying mechanisms linking HNC to neurodegenerative processes, which warrants further investigation. Understanding these mechanisms could lead to improved preventive and therapeutic approaches for managing both conditions.

### 3.4. Subgroup Analysis

Patients with HNC aged 55 to 69 demonstrated a higher risk ratio for developing PD compared to those aged 70 years or older (Table 4). To further analyze the risk of PD based on the type of HNC, the cancer groups were categorized into oral cavity, salivary gland, oropharynx, nasopharynx, hypopharynx, sinonasal tract, and larynx. Results from univariate and multivariate Cox regression models indicated that the adjusted HR for PD events in the oral cavity cancer subgroup was 1.93 (95% CI = 1.05–3.55). Although statistically significant results were not obtained for all subgroups, the adjusted HRs were notably higher in some cases. Specifically, the adjusted HR for PD was 3.70 (95% CI = 0.51−27.04) in the oropharynx cancer and 4.80 (95% CI = 0.65−35.29) in the nasopharynx cancer groups, both showing higher HRs than other cancer groups, such as salivary gland, hypopharynx, sinonasal tract, and larynx (Table 5). These findings suggest that certain types of HNC may be associated with a higher risk of developing PD, particularly in younger patients within the specified age range.

## 4. Discussion

Cancer results from DNA damage or mutations that disrupt the regulation of normal cell growth and division, thereby contributing to the development of various diseases through multiple pathways. Recently, there has been increasing interest in the association between cancer and the development of neurodegenerative diseases, which can significantly affect patients’ quality of life and lead to psychosocial issues. In particular, numerous studies have focused on the relationship between cancer and neurodegenerative movement disorders such as PD [4,5,6,7,9,10]. Several studies have investigated the relationship between PD and various types of cancer, such as colorectal, skin, lung, and prostate cancers, and have shown conflicting views of inverse and positive associations depending on the type of cancer [4,6,11]. However, the evidence regarding the association between HNC and PD remains insufficient. 

To the best of our knowledge, the present study was the first to analyze the risk of developing PD in patients with HNC using a representative nationwide population-based cohort dataset. We found that the incidence of PD in patients with HNC was significantly higher than in the non-cancer group. Furthermore, the HR of PD after adjusting for sex, age, residence, income level, and comorbidities was significantly higher in HNC group than in the non-cancer group (adjusted HR = 1.89, 95% CI = 1.08 to 3.33). Additionally, an analysis of the risk of developing PD by HNC subtype revealed that the oral cavity cancer group, one of the most common HNC types, had a significantly higher HR compared to the non-cancer group. Although the number of patients with other cancer types was too small to draw significant results, oropharyngeal and nasopharyngeal cancers showed higher HRs than the other types. 

However, the exact mechanisms by which HNCs influence the pathogenesis of PD remain unclear. Nevertheless, accumulating evidence from several studies suggests a potential association between HNC and PD, based on various aspects. Genetic factors may play a significant role in the potential link between HNC and PD. Mutations in the parkin gene are known to cause PD, primarily through their role in the ubiquitin–proteasome system, where they help degrade misfolded proteins, thereby preventing neurodegeneration [12,13]. Additionally, parkin functions as a tumor suppressor gene, and mutations in parkin can lead to the development of HNC such as nasopharyngeal carcinoma [14,15]. This dual role of parkin in neurodegeneration and tumor suppression highlights its potential involvement in the association between HNC and PD. However, some studies have suggested that genes crucial to the pathogenesis of HNC may play opposing roles in the development of PD. For instance, p53, a well-known tumor suppressor gene, is closely associated with the development of various cancers, including HNC, when mutated [16]. By contrast, in PD, p53 overexpression contributes to increased oxidative stress and mitochondrial dysfunction, leading to the death of dopaminergic neurons [17]. These contrasting roles of p53 underscore the complex interplay between HNC and PD. Understanding these genetic and molecular mechanisms is crucial to elucidate the relationship between these two conditions. Therefore, to clearly understand these heterogeneous genetic and molecular mechanisms of PD, future studies should focus on the impact of cancer risk in patients with genetic forms of PD.

Additionally, the treatment methods for HNC can influence the development of PD. Primary treatment strategies for HNC include surgery alone, postoperative radiation therapy, and radiation therapy alone. Radiotherapy is administered to almost all patients to improve their survival rates. Specifically, oral cavity cancer, a subtype that demonstrated a significantly higher HR among HNCs in our study, poses a risk of radiation-related brain damage owing to the proximity of the treatment site to the brain. This close anatomical relationship increases the likelihood of radiation-induced brain damage, potentially contributing to the development of PD. One case report described the development of secondary PD in a patient who underwent chemoradiotherapy for low-grade astrocytoma, suggesting that radiation near the substantia nigra can lead to damage and subsequent degeneration of dopaminergic neurons [18]. Moreover, one cohort study investigated the effects of radiation exposure on the risk of PD among individuals with long-term occupational exposure to external radiation and reported that such exposure could increase the risk of developing PD. This study emphasized that even low-dose radiation exposure contributes significantly to the development of neurodegenerative diseases [19]. Various in vitro studies have elucidated the mechanisms by which radiation exposure contributes to PD pathogenesis. Radiation exposure induces numerous irreparable and lethal DNA lesions, resulting in significant genomic instability. The cellular response to radiation involves the production of reactive oxygen species and spontaneous hydrolytic reactions, which exacerbate DNA damage. These processes create a conducive environment for the early apoptosis of PD neuronal cells [20]. Furthermore, radiation interacts with mitochondrial DNA and generates reactive hydroxyl radicals, leading to mitochondrial dysfunction [21]. Mitochondrial impairment results in an imbalance in energy and calcium homeostasis, inducing neuronal cell death [22]. Consequently, mitochondrial defects contribute to neurodegeneration, particularly during the early stages of PD pathogenesis [23]. Previous studies have shown that radiation therapy for HNC significantly contributes to the development of PD.

Complications associated with surgery for HNC can also influence the development of PD. Compared to other cancers, HNC often results in significant impairment of the ability to speak or eat, causing substantial inconvenience in daily life and severe cosmetic issues, which can be particularly stressful for patients. Consequently, patients with HNC experience higher levels of anxiety and depression than those with other types of cancer [24,25]. Several studies have reported that depression has a positive association with the development of PD [26,27,28,29,30]. One cohort study found that patients diagnosed with depression had a significantly increased risk of developing PD, with a relative risk of 3.13. This study indicated that the risk of PD was particularly elevated during the initial years following a diagnosis [27]. Furthermore, some studies have reported that peripheral inflammatory agents increased by depression can cross the blood–brain barrier and induce glial cell activation and that the activated glial cells cause neuroinflammation, which, through oxidative stress and glutamate excitotoxicity, leads to neurodegeneration and contributes to the pathogenesis of PD [31,32,33]. These findings imply a connection between depression, chronic stress, and PD, indicating that chronic stress and depression experienced by patients with HNC and surgery may play a role in the pathogenesis of PD.

The strengths of this study are as follows. First, this was the first cohort study to assess the incidence and risk of PD in Korean patients with HNC using nationwide population-based data. The health insurance claims databases used were comprehensive and built from data accumulated through the health insurance system, providing valuable information on health promotion, prevention, diagnosis, treatment, rehabilitation, childbirth, and mortality. To control for potential confounding factors, we matched sexual behavior scores, ensuring the comparability of incidence rates between the two groups. However, the retrospective nature of this cohort study limited our ability to definitively establish whether the observed associations were novel or coincidental. Despite these limitations, our results have significant clinical implications for patient care in the real-world setting. Second, this study was strengthened by the inclusion of a substantial number of patients and a long follow-up period of 10 years. The cohort database encompasses extensive information on medical visits, including outpatient and inpatient data, which is crucial for enhancing the power of our analysis. Finally, this nationwide cohort was based on claims data and included detailed sociodemographic, healthcare utilization, health-screening, and healthcare provider information. Adjusting for these sociodemographic characteristics helps minimize errors related to surveillance bias when assessing the risk of PD in patients with HNC. Collectively, these strengths improve the reliability and validity of our findings.

However, this study has several limitations that necessitate careful interpretation of the results. First and foremost, patients with HNC and PD were identified solely based on ICD-10 diagnostic codes. These codes do not provide detailed medical records, including comprehensive information on medical history and pathological reports. Consequently, it was impossible to analyze important clinical details such as the stage of cancer, specific pathology, and detailed aspects of PD. This lack of granularity limits our ability to understand the nuances of the diseases under investigation fully. Secondly, our study could not access information regarding the treatment methods for HNC. As a result, we were unable to assess how different cancer treatment modalities, such as chemotherapy and radiotherapy, and their durations might influence the risk of developing PD. This represents a significant gap, as treatment methods can have varying impacts on patient outcomes and the progression of comorbid conditions. Without this data, our findings regarding the relationship between HNC and PD are less comprehensive. Additionally, due to de-identification protocols, the database only provided age data in grouped categories rather than specific ages. To address this, we matched the two groups by categorizing their age data, but this approach may have introduced residual bias into our analysis. Age is a critical factor in the progression and onset of diseases, and the lack of precise age information means that some subtle but significant differences could have been overlooked. Moreover, we did not fully control for other neurodegenerative diseases that could influence the onset of PD. Thus, we cannot completely rule out the possibility that other neurodegenerative diseases associated with HNC may have impacted the development of PD. Thirdly, in the analysis of the HNC subtype, the small number of patients included and the limited PD events across each HNC subtype, except for the oral cavity cancer group, restrict the ability to derive significant clinical implications from the data through statistical analysis. Moreover, as this was a retrospective study, we faced inherent limitations associated with this study design. Retrospective studies rely on previously collected data, which can lead to issues such as incomplete records or lack of control over variables. Importantly, the retrospective nature of the study means that the pathological mechanisms underlying HNC and PD could not be directly investigated and analyzed. Understanding these mechanisms requires prospective clinical studies that can collect a wider range of factors in real time. Future research should aim to overcome these limitations by incorporating detailed medical records, including patient history and pathology reports, to provide a more comprehensive understanding of the diseases. Prospective studies are necessary to examine the effects of various treatment methods for HNC and their impact on the risk of PD. Additionally, obtaining precise age data rather than grouped categories would enhance the accuracy of age-related analyses. Finally, clinical studies designed to investigate the pathophysiological mechanisms in greater detail are essential to elucidate the underlying biological interactions between HNC and PD.

## 5. Conclusions

This study explored the potential link between HNC and the onset of PD. Our findings revealed that patients with HNC have an elevated risk of developing PD, with a particularly strong association observed in patients with oral cavity cancer. These results provide new insights into the relationship between HNC and PD. Consequently, clinicians should remain vigilant about the possible emergence of PD in patients with HNC, monitor any movement disorders and physical symptoms, and refer patients to a neurologist when necessary.

## Figures and Tables

**Table 1:** Characteristics of the study subjects.

Variables	Control (*n* = 2296)	Head and Neck Cancer (*n* = 574)	*p*-Value
Sex			1.000
Male	1072 (46.7%)	268 (46.7%)	
Female	1224 (53.3%)	306 (53.3%)	
Ages (years)			1.000
55–69	1280 (55.7%)	320 (55.7%)	
>69	1016 (44.3%)	254 (44.3%)	
Residence			1.000
Seoul	300 (13.1%)	75 (13.1%)	
Second area	364 (15.9%)	91 (15.9%)	
Third area	1632 (71.1%)	408 (71.1%)	
Household income			1.000
Low (0–30%)	616 (26.8%)	154 (26.8%)	
Middle (30–70%)	688 (30.0%)	172 (30.0%)	
High (70–100%)	992 (43.2%)	248 (43.2%)	
CCI			1.000
0	1244 (54.2%)	311 (54.2%)	
1	576 (25.1%)	144 (25.1%)	
≥2	476 (20.7%)	119 (20.7%)	

Comparison of subjects without cancer; Seoul, the largest metropolitan area; second area, other metropolitan cities; third area, other areas; CCI, Charlson comorbidity index.

**Table 2:** Incidence and risk of incident Parkinson’s disease.

Variables	N	Cases	Person-Years	Incidence Rate	Unadjusted HR (95% CI)	Adjusted HR (95% CI)
Parkinson’s disease						
Comparison	2296	42	19,301.1	2.18	1.00 (ref)	1.00 (ref)
Head and Neck Cancer	574	17	4077.7	4.17	1.90 (1.08–3.34) *	1.89 (1.08–3.33) *

HR, hazard ratio; CI, confidence interval. * *p* < 0.05.

**Table 3:** Risk of Parkinson’s disease in head and neck cancer group by time.

Time (Year)	Number of Events		Adjusted HR (95% CI)
	Comparison	Head and Neck Cancer	
1	2	1	1.98 (0.18−21.89)
2	6	3	2.03 (0.51−8.13)
3	11	6	2.27 (0.84−6.14)
4	17	7	1.74 (0.72−4.21)
5	20	8	1.71 (0.75−3.89)
6	22	10	1.97 (0.93−4.16)
7	25	13	2.27 (1.16−4.44) *
8	34	15	1.97 (1.07−3.62) *
9	40	17	1.97 (1.11−3.48) *
10	41	17	1.93 (1.10−3.41) *
11	42	17	1.89 (1.08−3.33) *

HR, hazard ratio; CI, confidence interval. * *p* < 0.05.

**Table 4:** Hazard ratios of Parkinson’s disease by age between comparison and cancer group.

Age	55−69		>69	
	Comparison	Head and Neck Cancer	Comparison	Head and Neck Cancer
Parkinson’s disease				
Unadjusted HR (95% CI)	1.00 (ref)	2.70 (1.24−5.84) *	1.00 (ref)	1.33 (0.57−3.09)
Adjusted HR (95% CI)	1.00 (ref)	2.74 (1.26−5.95) *	1.00 (ref)	1.33 (0.57−3.10)

HR, hazard ratio; CI, confidence interval. * *p* < 0.05.

**Table 5:** Incidence and risk of incident Parkinson’s disease event according to the subtype of head and neck cancer.

Variables	N	Cases	Person-Years	Incidence Rate	Unadjusted HR (95% CI)	Adjusted HR (95% CI)
Cancer type						
Comparison	2296	42	19,301.1	2.18	1.00 (ref)	1.00 (ref)
Oral cavity	423	14	3183.3	4.40	2.00 (1.09−3.66) *	1.93 (1.05−3.55) *
Salivary gland	8	0	45.3	-	N/A	N/A
Oropharynx	21	1	134.4	7.44	3.43 (0.47−24.90)	3.70 (0.51−27.04)
Nasopharynx	23	1	124.6	8.03	3.69 (0.51−26.80)	4.80 (0.65−35.29)
Hypopharynx	8	0	46.3	-	N/A	N/A
Sinonasal tract	7	0	24.1	-	N/A	N/A
Larynx	84	1	519.6	1.92	0.89 (0.12−6.45)	1.05 (0.14−7.79)

HR, hazard ratio; CI, confidence interval; N/A, no applicable; * *p* < 0.05.

## Data Availability

The datasets generated and/or analyzed in the current study are not publicly available owing to the policy of the Korea National Health Insurance Service but are available from the corresponding author upon reasonable request.
